# Linkage to HIV care and early retention in HIV care among men in the ‘universal test-and-treat’ era in a high HIV-burdened district, KwaZulu-Natal, South Africa

**DOI:** 10.1186/s12913-024-10736-3

**Published:** 2024-04-02

**Authors:** Mbuzeleni Hlongwa, Wisdom Basera, Khumbulani Hlongwana, Carl Lombard, Ria Laubscher, Sinegugu Duma, Mireille Cheyip, Debbie Bradshaw, Edward Nicol

**Affiliations:** 1 Burden of Disease Research Unit, South African Medical Research Council, Cape Town, South Africa; 2 School of Nursing and Public Health Medicine, University of KwaZulu-Natal, Durban, South Africa; 3 Public Health, Societies and Belonging, Human Sciences Research Council, Pretoria, South Africa; 4 School of Public Health and Family Medicine, University of Cape Town, Cape Town, South Africa; 5 Cancer & Infectious Diseases Epidemiology Research Unit, University of KwaZulu-Natal, Durban, South Africa; 6 South African Medical Research Council, Biostatistics, Cape Town, South Africa; 7 Division of Global HIV&TB, Centers for Disease Control and Prevention, Pretoria, South Africa; 8 Division of Health Systems and Public Health, Stellenbosch University, Cape Town, South Africa

**Keywords:** HIV treatment, Men, Linkage to care, Retention in care, South Africa

## Abstract

**Introduction:**

Despite the numerous efforts and initiatives, males with HIV are still less likely than women to receive HIV treatment. Across Sub-Saharan Africa, men are tested, linked, and retained in HIV care at lower rates than women, and South Africa is no exception. This is despite the introduction of the universal test-and-treat (UTT) prevention strategy anticipated to improve the uptake of HIV services. The aim of this study was to investigate linkage to and retention in care rates of an HIV-positive cohort of men in a high HIV prevalence rural district in KwaZulu-Natal province, South Africa.

**Methods:**

From January 2018 to July 2019, we conducted an observational cohort study in 18 primary health care institutions in the uThukela district. Patient-level survey and clinical data were collected at baseline, 4-months and 12-months, using isiZulu and English REDCap-based questionnaires. We verified data through TIER.Net, Rapid mortality survey (RMS), and the National Health Laboratory Service (NHLS) databases. Data were analyzed using STATA version 15.1, with confidence intervals and *p*-value of ≤0.05 considered statistically significant.

**Results:**

The study sample consisted of 343 male participants diagnosed with HIV and who reside in uThukela District. The median age was 33 years (interquartile range (IQR): 29–40), and more than half (56%; *n* = 193) were aged 18–34 years. Almost all participants (99.7%; *n* = 342) were Black African, with 84.5% (*n* = 290) being in a romantic relationship. The majority of participants (85%; *n* = 292) were linked to care within three months of follow-up. Short-term retention in care (≤ 12 months) was 46% (*n* = 132) among men who were linked to care within three months.

**Conclusion:**

While the implementation of the UTT strategy has had positive influence on improving linkage to care, men’s access of HIV treatment remains inconsistent and may require additional innovative strategies.

## Introduction

Despite progress in the proportion of people living with HIV (PLHIV) and receiving treatment in sub-Saharan Africa (SSA), significant gender inequities still exist [1]. Men living with HIV remain less likely than women to access HIV care, despite the many efforts and interventions implemented [2]. Men are tested, linked, and retained in HIV services at lower rates than women across SSA [1]. When men are diagnosed with HIV and put on treatment, they are more likely than their female counterparts to have frequent treatment disruptions [3]. Antiretroviral treatment (ART) is widely available and accessible to eligible people in South Africa; however, some factors prevent eligible men from accessing HIV treatment services in public health care facilities. These factors include dominant masculine norms, the design of health services, long queues in health care facilities, stigma associated with HIV, perceived potential breach of confidentiality regarding one’s HIV status, long waiting times at health care facilities, and money and time spent traveling to seek care [4, 5]. Linkage to and retention in care are critical in achieving the 95–95–95 targets (95% of all people living with HIV should be diagnosed, 95% of people diagnosed with HIV are started on ART, and 95% of people started on ART have a suppressed viral load) [6]. However, retaining men in HIV treatment remains one of the most difficult challenges of this intervention programme.

The expansion of HIV testing, and treatment has yielded better outcomes in women more than in men. Lower HIV testing rates, as well as poor linkage to and retention on antiretroviral therapy (ART), may be contributing to the growing female-male discrepancy in adult life expectancy [1]. In comparison to women, men are diagnosed with HIV and put on ART at an advanced stage [7]. Men are more likely than women to have virological failure as a result of frequent treatment interruptions, resulting in higher HIV-related death rates, while on ART [8, 9]. To improve health outcomes, reduce HIV transmission rates, and achieve epidemic containment, it is critical to understand the barriers and enablers to HIV treatment among men in resource-limited settings.

The ‘Universal Test-and-Treat’ (UTT) strategy was introduced to ensure that all individuals diagnosed with HIV are started on ART as soon as possible, regardless of CD4 count or clinical staging [10]. It also refers to preferably same day initiation of ART but within 14 days of initial HIV-positive diagnosis at the maximum. Since 2016, South Africa has been implementing the UTT strategy in all public health care facilities [11]. However, more data is required to understand how the adoption of the UTT in South Africa has affected the linkage to and retention in care, particularly among men — a population that is often viewed as “hard to reach”. Prior to the launch of the UTT strategy, Haber et al.’s study revealed that less than half of the study participants in the KwaZulu-Natal province were linked to HIV treatment [12].

Linkage to and retention in care among HIV diagnosed men are key to improving the health and well-being of men infected with HIV. These factors remain top priorities in South Africa, as part of efforts by the government to curb the spread of HIV as well as reaching the 95-95-95 targets. Linkage to and retention in HIV treatment are crucial for attaining viral load reduction in populations and, thus, reducing HIV transmission [13]. To better understand the impact of UTT on HIV treatment cascade, we assessed the linkage to and retention in care rates of an HIV-positive cohort of men in a high HIV burdened rural district in KwaZulu-Natal province, South Africa.

## Methods

### Study setting

**Fig. 1:**
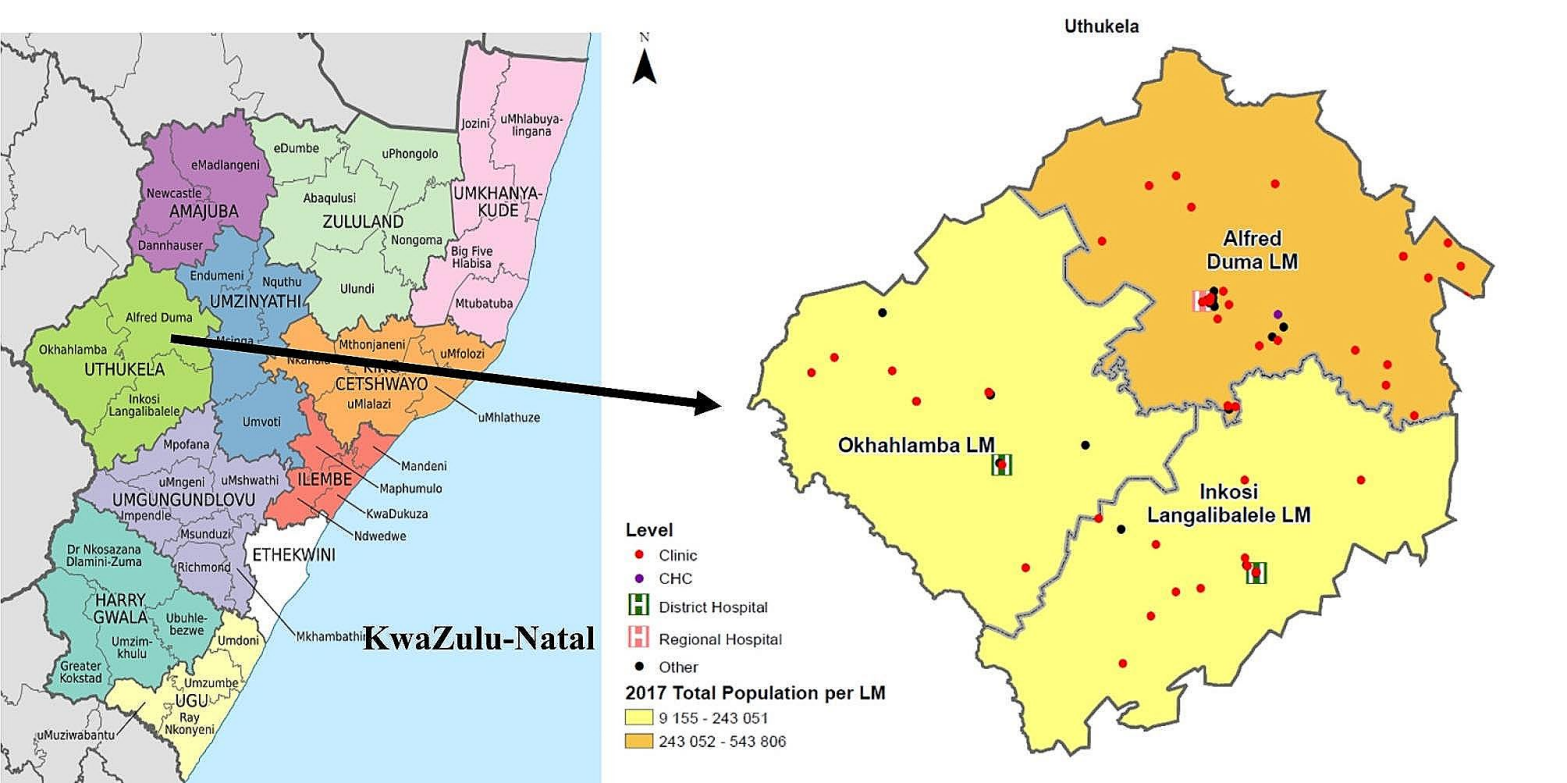
Map of uThukela District, KwaZulu-Natal, 2017, showing the population density for the three local municipalities LM: local municipality; CHC: Community Health Centre

This study was conducted in the uThukela District Municipality in KwaZulu-Natal. The district is comprised of three local municipalities (LMs) - Alfred Duma LM, Inkosi Langalibalele LM and Okhahlamba LM (Fig. 1). The population of uThukela district is predominantly poor, rural and utilizing public health services [14]. HIV prevalence is high in the district at 22% among men aged 15–49 years, with individuals required to travel long distances to access basic health care services [14, 15].

### Study design

An observational cohort design was undertaken in 18 healthcare facilities in uThukela district.

### Sample size determination and sampling strategy

We undertook a sample size calculation based on the primary outcome, which was the proportion of individuals who were linked to HIV care. Successful linkage to care was defined as proportion for participants from the enrolled facilities who were diagnosed with HIV and subsequently completed a first medical clinic visit and started on ART within 90 days of their positive HIV test at enrollment as evidenced by a TIER.Net record, an electronic patient management system capturing patient-level data on public sector HIV management in South Africa [16]. At the time of our data collection, the uThukela district did not have published data on linkage to and retention in care rates among men during the UTT era. However, a linkage to care rate of 62% within the first year of HIV diagnosis has been reported from KwaZulu-Natal province, South Africa [8]. Therefore, we hypothesized a 10% increase in linkage to care rate due to the possible impact of the UTT strategy.

The sample size calculation (Table 1) for this analysis are illustrative of the power of the design of the study to detect a difference in the potential influence of UTT on the linkage to care rates at a specific time point (i.e., 3 months). The minimum number of clusters (facilities) needed to detect an increase in linkage to care uptake at 3 months with 76% power was 18 in total, translating to 9 for the two time periods i.e., baseline and follow-up with 22 participants per cluster assuming an interclass correlation (ICC) of 0.01 and significance level of 0.05. The sample and power calculation were done using STATA v14.2 (StataCorp, Texas, USA). The study had the capacity to retain at least 70% power for a 20% dropout at 3 months of follow-up (m = 18 per cluster) and the design effect of 1.2 were deemed realistic for the study populations.

**Table 1:** Power and sample size calculation in the male linkage to care study in uThukela District, KwaZulu-Natal, 2019

p_1_	p_2_	delta	alpha	Beta	k_1_	k_2_	m_1_	m_2_	rho
0.62	0.72	0.10	0.05	0.76	9	9	22	22	0.01

Where *p - linkage to care proportions; delta– difference between proportions; alpha - z values used for calculating type 1 error; beta - z value used for calculating power; rho– inter-cluster correlation; m– average cluster size and k - number of clusters. N - sample size for study period allowing for cluster randomization*

There are 63 public sector health care facilities providing primary health services in uThukela district. To ensure feasibility of reaching the enrolment targets, a threshold was set to include only facilities which reported 10 or more patients testing positive per month (average over 12 months, December 2016– November 2017, DHIS data). The study selected 18 health care facilities based on the HIV testing uptake rates. These facilities, which include three gateway clinics, eight primary health care (PHC) clinics, two community day centers, three mobile clinics, one community health center (CHC), and an outpatient department within a hospital, were likely to yield the required minimum number of tests to increase our chances of enrolling 10 participants per day. We assumed a conservative number of five male participants to be enrolled from the target 18 facilities each month equaling to 90 participants over a period of six months, yielding a possible 540 participants - which would cover the required sample size of 396 (22 × 18) (Table 1). We adopted a convenience sampling of study participants until we reached the targeted number of participants.

Adults 18 years and older visiting different facilities to conduct an HIV test were approached to consider participating in the study after it had been fully introduced to them. Those who consented to participating in the study were requested to complete a self-administered questionnaire at baseline, before testing for HIV, and a follow-up at 4 months post HIV diagnosis.

Our inclusion criteria for enrolment into the study involved participants aged 18 years or older; intending to take an HIV test in one of the participating health care facilities during the study period; access to a cell phone and willingness to provide contact details. A detailed methodology is described elsewhere [17].

### Definitions

We defined linkage to HIV care in this analysis as the successful completion of a first medical clinic visit after HIV diagnosis within three months after HIV-positive diagnosis, and have been initiated onto ART as verified through TIER.Net record. Retention in care was defined as the proportion of HIV-positive participants who were linked on ART, remained in contact with HIV care services and are active on ART, and were not reported as dead or having interrupted treatment during the last 12 months post HIV diagnosis.

### Data collection and management

Data were collected using isiZulu and English REDCap-based questionnaires, as well as through record reviews. While prospective participants were waiting in the HIV testing queues, fieldworkers invited them to enroll in the study. Each questionnaire was completed within 45–60 min, depending on whether it was self-administered or completed with assistance. The survey instrument included questions on participants’ demographic and socio-economic characteristics, HIV testing and care experiences, and HIV disclosure. At the end of each day, participants’ HIV test results were retrieved from clinic records for verification. The Tier.Net database and the NHLS database were used to track participants who tested positive as they interacted with the health care system for CD4, ART and viral load measurements. We also used the NHLS’ central data warehouse (CDW) probabilistic linkage algorithm to link the results for participants using names, dates of birth, sex, initial laboratory identification, etc. Also, the presence and timing of each participant’s most recent viral load measurements obtained from TIER.Net/NHLS was considered for retention in care.

After data was retrieved from the TIER.Net and NHLS databases, we de-identified participants’ records and entered them into the REDCap-based participant questionnaires using the unique enrolment ID number before extracting them for cleaning and analysis using STATA version 14.2 (StataCorp, Texas, USA). Participants’ mortality status was checked at the end of the 12 months follow-up via the South African Medical Research Council (SAMRC) rapid mortality survey (RMS) database to explain their treatment interruption status and confirm that individuals considered as not linked to care or not retained in care were not deceased. The RMS database contains monthly information about deaths registered by the South African Department of Home Affairs.

### Statistical analysis

Data were analyzed using the svyset command in STATA 14.2 (StataCorp, Texas, USA) to incorporate the one stage cluster study design of the sample. The primary sampling unit was the name of the facility which represented the cluster were the survey participants were sampled from stratified by the type of facility. There was no finite population correction since the number of possible participants was not known beforehand. Once the survey setting was declared, the categorical participant characteristics were reported as proportions and the associated 95% confidence intervals (CIs). Continuous data were reported as medians, interquartile range (IQR) since the data were skewed. Linkage to and retention in care were expressed as proportions of the cohort living with HIV. The Mann-Whitney test was used to investigate differences between numerical variables and the Pearson’s chi-squared test which was used to assess associations between categorical variables was corrected using the second order correction of Rao and Scott and converted into an F statistic. Confidence intervals, the test statistic (F) and a *p*-value of ≤0.05 were reported to consider statistical significance for the various characteristics of the sample.

### Ethical considerations

Approvals to conduct this study were obtained from the SAMRC ethics committee. This project was reviewed in accordance with CDC human research protection procedures and was determined to be research involving human subjects, but CDC investigators did not interact with human participants or have access to identifiable data or specimens for research purposes. Additional permissions to conduct this study were obtained from the KZN Provincial Department of Health and the uThukela district authorities. Before taking part in the study, all participants signed an informed consent form. We used research pseudonyms instead of participant names to maintain confidentiality and privacy of our participants. Data was stored at SAMRC in a password protected file. Referral pamphlets with details of support centers were made available to participants who would have become emotionally distressed during data collection.

## Results

**Fig. 2:**
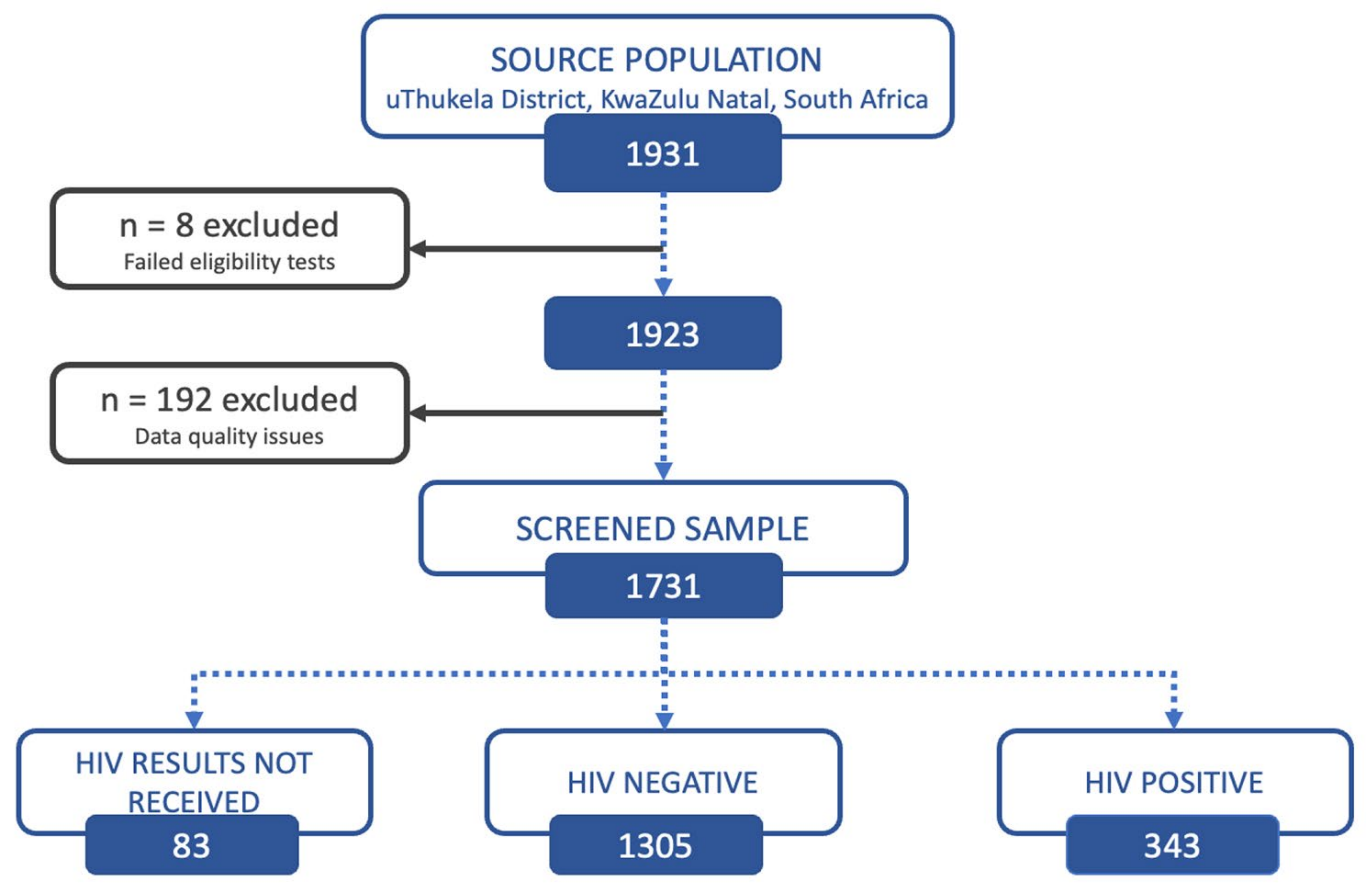
Consort diagram detailing the recruitment of the male participants into the Linkage to Care study in uThukela district, 2018

A total of 1731 male participants were recruited after the screening of 1931 consenting participants approached for inclusion eligibility in the study. A small proportion was excluded because they failed eligibility checks (0.4%; *n* = 8) and others had data quality issues (9.9%; *n* = 192) including enrolment ID duplicates, incomplete questionnaires, or no enrolment numbers. Of those recruited via screening for HIV reactivity (*n* = 1731), a small proportion did not receive their test results (4.8%; *n* = 83), most were HIV negative (75.4%; *n* = 1305) and almost a quarter were HIV positive (19.8%; *n* = 343). The final study sample for this analysis consisted of 343 male participants who were residing in the uThukela District from December 2017 to June 2018 (Fig. 2).

The median age was 33 years (IQR: 29–40) and more than half (56%; *n* = 193) were aged 18–34 years. Almost all participants (99.7%; *n* = 342) were Black African, with the majority (98.0%; *n* = 336) being South African citizens. In terms of marital status, 84.5% (*n* = 290) of participants were in a romantic relationship (married - living together; cohabiting; dating), 1.7% (*n* = 6) were married but not living together with their partner, and 13.7% (*n* = 47) were single. More than half (60.2%; *n* = 204) had completed a secondary level of education, 5.6% (*n* = 19) had completed a primary level of education, and 2.4% (*n* = 8) had no education at all (Table 2).

### Linkage to HIV care

The majority of men, 84.8% (*n* = 292) were linked to HIV treatment within three months of follow-up and could be tracked in the TIER.Net database and 15.2% (51) were identified as not having linked to care at the time (Table 2). Of the participants diagnosed with HIV at a clinic and hospital settings, 80.4% (*n* = 189) and 100% (*n* = 38) were linked to HIV treatment, respectively. Linkage to care was high regardless of the level of education, ranging from 83.3% (*n* = 170) among participants who had completed secondary level of education to 89.5% (*n* = 17) among those who completed primary level of education. Similarly, linkage to care was high across all age categories, ranging from 81.3% (*n* = 26) among participants aged 18–24 years to 91.7% (*n* = 22) among participants aged 50 years and older. Linkage to care was high regardless of participants’ marital status, it was 82.6% (*n* = 57) for those who were cohabitating, 84.1% for those dating and 89.4% (*n* = 42) among those who were single. Participants who were married and living together with their partner accounted for 90.6% (*n* = 29) compared to 83.3% (*n* = 5) among those who were married and living separately from their partner. The variable facility type (F = 1.5 and *p* = < 0.01) was a significant predictor for linkage to care.

**Table 2:** Demographic characteristics of participants disaggregated by linkage to HIV care within three months of follow-up, uThukela District, KwaZulu-Natal, 2019

Variable	Total (*N* = 343)			Not linked in care (*N* = 51)			Linked in care (*N* = 292)				
	n	%	95% CI	N	%	95% CI	n	%	95% CI	F-value	*p*-value
**Nationality**											
South African citizen Other SADC Other African	336 6 1	98.0 1.7 0.3	95.6–99.1 0.7–4.6 0.0-2.5	51 0 0	15.2 0 0	11.4–20.0 - -	285 6 1	84.8 100 100	80.9–88.6 - -	0.5	0.58
**Facility type**											
Clinics Gateway Hospital Mobile clinic	235 64 38 6	68.5 18.7 11.1 1.7	56.7–78.3 12.7–26.7 4.6–24.2 0.2–13.4	46 5 0 0	19.6 7.8 0 0	14.7–25.6 4.5–13.2 - -	189 59 38 6	80.4 92.2 100 100	74.4–85.3 86.8–95.5 - -	1.5	***< 0.01***
**Ethnicity**											
Black African Colored/Mixed ancestry	342 1	99.7 0.3	97.6–99.9 0.0-2.4	51 0	14.9 0	11.2–19.6 -	291 1	85.1 100	80.4–88.8 -	0.6	0.73
**Education level**											
No education Primary education Secondary education Post matriculation	8 19 204 108	2.4 5.6 60.2 31.9	0.9–5.8 3.6–8.7 53.3–66.6 25.2–39.4	1 2 34 13	12.5 10.5 16.7 12.0	1.6–55.3 2.4–35.8 12.6–21.7 5.9–23.1	7 17 170 95	87.5 89.5 83.3 88.0	44.7–98.4 64.2–97.6 78.3–87.4 76.9–94.1	0.5	0.61
**Age, median (IQR)**	343	33	29–40	51	33	29–38	292	33	29–40	-0.4	0.68 *
**Age categories**											
18–24 years 25–29 years 30–34 years 35–49 years 50 + years	32 69 92 126 24	9.3 20.1 26.8 36.7 7.0	6.2–13.9 14.6–27.0 20.9–33.7 29.6–44.5 3.5–13.4	6 9 17 17 2	18.8 13.0 18.5 13.5 8.3	17.1–36.4 6.1–23.3 11.1–27.9 8.1–20.7 1.0–27.0	26 60 75 109 22	81.3 87.0 81.5 86.5 91.7	67.5–90.0 75.0-93.7 72.7–88.0 81.1–90.5 71.3–98.0	0.9	0.46
**Marital status**											
Married (living together) Married (living separately) Cohabiting Dating Single	32 6 69 189 47	9.3 1.7 20.1 55.1 13.7	5.7–15.3 0.7–3.9 16.5–25.5 51.2–62.1 7.9–15.0	3 1 12 30 5	9.4 16.7 17.4 15.9 10.6	2.7–27.5 4.4–46.4 8.8–31.5 10.9–24.1 3.5–23.1	29 5 57 159 42	90.6 83.3 82.6 84.1 89.4	72.5–97.3 53.6–95.6 68.5–91.2 75.9–89.9 76.9–96.5	0.7	0.56

*p*-value of$\le 0.05$ was considered statistically significant

*p*-values derived using Mann Whitney U-test for continuous data

*p*-values derived using Pearson’s Chi-squared test considered the one stage cluster design

proportions (%) for the columns reported as n/N, the associated test statistic and the associated 95% CI

IQR– Interquartile range; CI– Confidence Interval

* z value reported.

### Retention in HIV care

Among the 292 males who were linked to care, 45.6% (*n* = 132) were retained in care after 12 months of follow-up (Table 3). The median age was 34 years (IQR: 29–41). Of the participants who were linked to care from a clinic and hospital settings, 42.3% (*n* = 80) and 65.8% (*n* = 25) were retained in care after 12 months of follow-up, respectively. Retention in care was 57.1% (*n* = 4) and 50.5% (*n* = 48) among participants who were not educated at all and those with a tertiary level of education, respectively. Among participants aged 18–24 years who were linked to care, 50.0% (*n* = 13) were retained in care after 12 months of follow-up. About half of participants who were cohabitating (50.9%; *n* = 29) were retained in care after 12 months of follow-up. Those who were married and living with their partners (*n* = 13) had the least proportion (44.8%) of retention in care.

**Table 3:** Demographic characteristics of participants disaggregated by whether they were retained in care after 12 months of follow-up, uThukela District, KwaZulu-Natal, 2019

Variable	Total (*n* = 292)			Retained in care (*n* = 132)			Not retained in care (*n* = 160)				
	N	%	95% CI	N	%	95% CI	N	%	95% CI	F-value	*p*-value^+^
**Nationality**											
South African citizen Other SADC Other African	285 6 1	97.6 2.1 0.3	94.8–98.9 0.8–5.4 0.0-2.9	130 2 0	45.6 33.3 0	32.9–58.9 8.3–73.5 -	155 4 1	54.4 66.7 100	41.1–67.1 26.5–91.7 -	1.2	0.56
**Facility type**											
Clinic Gateway Hospital Mobile clinic	189 59 38 6	64.7 20.2 13.0 2.1	52.4–75.4 14.0-28.2 5.5–27.7 0.2–15.4	80 24 25 3	42.3 40.7 65.8 50.0	27.5–58.7 15.5–72.0 50.8–78.2 50.0–50.0	109 35 13 3	57.7 59.3 34.2 50.0	41.3–72.5 45.7–71.9 21.9–49.2 50.0–50.0	1.2	**0.04**
**Education level**											
No education Primary education Secondary education Tertiary	7 17 170 95	2.4 5.9 58.8 32.9	0.9–6.3 3.6–9.5 51.0-66.2 25.8–40.8	4 6 73 48	57.1 35.3 42.9 50.5	30.6–80.2 14.1–64.6 29.0-58.1 37.4–63.6	3 11 97 47	42.9 64.7 57.1 49.5	19.9–69.4 35.5–86.0 41.9–71.0 36.4–62.7	1.0	0.40
**Age in years**	292	33.0	29.0–40.0	132	34.0	28.0–41.0	160	33.0	29–40	0.1	0.92 *
**Age categories**											
18–24 years 25–29 years 30–34 years 35–49 years 50 + years	26 60 75 109 22	8.9 20.5 25.7 37.3 7.5	5.9–12.1 16.3–25.1 21.7–31.2 32.6–43.1 4.5–10.1	13 27 33 48 11	50.0 45.0 44.0 44.0 50.0	24.3–75.7 28.3–63.0 26.3–63.4 30.0-59.5 30.4–69.6	13 33 42 61 11	50.0 55.0 56.0 56.0 50.0	24.3–75.7 37.0-71.7 36.6–73.7 40.5–70.3 30.4–69.6	0.1	0.94
**Marital status**											
Married (living together) Married (living separately) Cohabiting Dating Single	29 5 57 159 34	10.2 1.8 20.1 56.0 12.0	6.5–15.6 0.7–4.6 14.2–27.6 50.5–61.4 7.2–19.3	13 4 29 92 18	44.8 80.0 50.9 57.9 52.9	20.0-72.6 39.1–96.2 34.4–67.2 44.2–70.2 33.9–71.2	16 1 28 67 16	55.2 20.0 49.1 42.1 47.1	27.4–80.1 3.9–61.0 32.8–65.6 29.8–55.6 27.8–66.2	0.9	0.44

*p*-value of$\le 0.05$ was considered statistically significant

*p*-values derived using Mann Whitney U-test for continuous data

*p*-values derived using Pearson’s Chi-squared test considered the one stage cluster design

proportions (%) for the columns reported as n/N, the associated test statistic and the associated 95% CI

IQR– Interquartile range; CI– Confidence Interval

* z value reported

## Discussion

In this study, we investigated the linkage to and retention in care rates of a cohort of men living with HIV in uThukela district, KwaZulu-Natal province, South Africa. Our results showed that linkage to care among males who were diagnosed with HIV was high, with 85% initiating HIV treatment within three months of knowing their HIV status. However, retention in HIV care after 12 months post diagnosis was low, at 45%.

Timely and substantial linkage to HIV treatment among men newly diagnosed with HIV is critical to improve health outcomes and maximize population-level benefits from ART. Despite the well documented barriers deterring men from accessing health services in SSA [4, 18], our study findings showed higher rates of linkage to care compared to findings from similar settings in KwaZulu-Natal and in Western Cape provinces [19, 20]. A household-based community cross-sectional survey conducted in KwaZulu-Natal province found that 71% (95% CI: 68.6–73.4) of participants were linked to care, accounting for 74% (95% CI: 71.6–76.9) among women and 60% (95% CI: 54.2–66.1) among men [19]. In Cape Town, Western Cape province, 63% of participants who were diagnosed with HIV were linked to care, with men accounting for 64% [20]. Furthermore, less than half of individuals diagnosed with HIV were linked to care in similar settings [12, 21, 22]. Our results suggest that the greatest challenge lies in men’s retention in care than their linkage to care.

Improving linkage to care is still a challenge in SSA [23]. However, our study was conducted during the era in which the UTT strategy was launched in South Africa, a fast-track strategy which allows for initiating all individuals testing HIV-positive on ART irrespective of their CD4 count and clinical staging [10]. The implementation of UTT has made important strides towards improving access to HIV treatment among individuals newly diagnosed with HIV [24]. Therefore, the high rates of linkage to care among men in our siting may be indicative of the success of the UTT strategy implementation. This is an important finding because it demonstrates the UTT programme success and effectiveness in engaging men into HIV treatment, while identifying the areas requiring further attention. Evidence suggests that early initiation on ART for individuals living with HIV is linked to better clinical outcomes than later treatment [25, 26].

Despite the benefits of linkage to care, retention in care is critical for continuous uptake of prescribed HIV treatment, to prevent morbidity and mortality resulting from treatment interruption among HIV-positive individuals [8]. Our finding that short-term retention in care was low is consistent with many reports from elsewhere in SSA [19, 27]. A study conducted in South Africa indicated that males (adjusted hazard ratio [aHR]: 1.48; 95% CI: 1.1–2.0) initiating ART under UTT were 50% more likely to be lost-to-follow up (LTFU) during the 12 months period after they were initiated in care [28]. Clearly there remains critical challenges deterring men from consistently accessing HIV treatment [4, 29]. Common risk factors of LTFU include financial constraints, dissatisfaction with health services and/or poor infrastructure [30–32]. Retaining men on consistent ART is important, given the increased virological failure and mortality rates associated with LTFU [8, 9]. While linkage to care is high in our study, the low retention rates suggests that more efforts are essential to improve retention in care among men, to improve health outcomes and curb the spread of HIV. Therefore, identifying men who are more likely to experience treatment interruptions may provide imperative prospects for designing tailored service delivery interventions which would be more responsive to their needs [33].

Our findings are subject to important strengths and limitations. Our determination of adherence to HIV treatment was confirmed via the availability of a medical clinic visit evidenced by a record in Tier.Net and the presence of viral load measurements in the NHLS database. The estimates from the study were, less precise as evidenced by the wide confidence intervals largely due to a small sample size. We could not collect information on the reasons for refusal to participate and the associated number of refusals as such the study sample could be subject to selection bias. Therefore, the study sample could be less representative of the source population and these results should be interpreted with caution. We did not measure treatment outcomes of participants during the 12-month period.

## Conclusion

Our findings illustrate that the adoption and implementation of the UTT strategy has had positive benefits towards improving linkage to care among men in the uThukela district. However, retention in care remains a concern given the low rates of men retained within 12 months of follow-up. These findings suggest the need for additional and targeted interventions to improve retention among men to address barriers deterring men from consistently accessing HIV treatment in healthcare facilities, to enhance their individual health outcomes, minimize HIV transmission, achieve epidemic control and extend life expectancy.

## Data Availability

All the data analysed and reported in this paper will be made available upon request. EN (edward.nicol@mrc.ac.za) should be contacted if someone wants to request the data from this study.
